# Evaluation of TGF-β1 and MCP-1 expression and tubulointerstitial fibrosis in children with Henoch-Schönlein purpura nephritis and IgA nephropathy: A clinical correlation

**DOI:** 10.6061/clinics/2017(02)05

**Published:** 2017-02

**Authors:** Zhao Shuiai, Shen Huijun, Gu Weizhong, Liu Aimin, Mao Jianhua

**Affiliations:** The Children–s Hospital of Zhejiang University School of Medicine, Department of Nephrology, Hangzhou 310003, Zhejiang Province, China

**Keywords:** Henoch-Schönlein Purpura Nephritis, IgA Nephropathy, Immunohistochemistry, Monocyte Chemoattractant Protein-1, Transforming Growth Factor β1, Fibrosis

## Abstract

**OBJECTIVES:**

Henoch-Schönlein purpura nephritis and immunoglobulin A nephropathy are two diseases with similar clinical presentations but very different prognoses. Transforming growth factor β1 and monocyte chemoattractant protein-1 have been associated with the development of tissue fibrosis. We examined the development of tubulointerstitial fibrosis and its relationship with Transforming growth factor β1 and monocyte chemoattractant protein-1 expression in these patients.

**METHODS:**

Renal tissue samples were collected by renal biopsy from 50 children with Henoch-Schönlein purpura nephritis and 50 children with immunoglobulin A nephropathy. Hematoxylin and eosin and Masson's trichrome-stained tissues were examined using light microscopy. Tubulointerstitial fibrosis was graded using the method described by Bohle et al. 1. The immunohistochemical detection of Transforming growth factor β1 and monocyte chemoattractant protein-1 expression was correlated with the tubulointerstitial fibrosis grade. Clinical Trial registration number: ZJCH-2012-0105.

**RESULTS:**

Transforming growth factor β1 and monocyte chemoattractant protein-1 expression in the renal tissues was significantly greater in the patients with immunoglobulin A nephropathy than in the patients with Henoch-Schönlein purpura nephritis (both *p*<0.001). The immunoglobulin A nephropathy patients had a higher tubulointerstitial fibrosis grade than the Henoch-Schönlein purpura nephritis patients (*p*<0.001). The tubulointerstitial fibrosis grade was in accordance with the Transforming growth factor β1 and monocyte chemoattractant protein-1 expression levels in both diseases (both *p*<0.001).

**CONCLUSION:**

Transforming growth factor β1 and monocyte chemoattractant protein-1 expression was associated with the development of immunoglobulin A nephropathy and Henoch-Schönlein purpura nephritis. Further studies are needed to better evaluate this association.

## INTRODUCTION

Henoch-Schönlein purpura (HSP) is one of the most common childhood vasculitides 2. Approximately 30 to 50% of children with HSP have nephritis (HSPN), which most commonly presents with hematuria and/or proteinuria. The microscopic findings of this disease include mesangial cell proliferation, crescent formation in epithelial cells, and mesangial IgA deposition 3. Most HSPN patients have a good prognosis 4. Although HSPN has been related to infection and allergies 5, the exact etiology and pathogenesis of this disease are unknown.

Immunoglobulin A nephropathy (IgAN) is the most common primary glomerular disease worldwide. IgAN is characterized by IgA or IgA immune complex deposition in the glomerular mesangial region with mesangial cell proliferation 6. The main clinical findings are isolated hematuria or proteinuria. A total of 15 to 20% of IgAN patients develop chronic renal failure within 10 years of their diagnosis. IgAN is one of the leading causes of end-stage renal disease in China 7. Although HSPN and IgAN share some manifestations 8, their prognoses are very different.

Tubulointerstitial damage is a key factor in determining the prognosis of IgAN patients 9,10. Tubulointerstitial damage in the setting of IgAN includes tubular epithelial cell degeneration, necrosis, or atrophy, interstitial inflammatory cell infiltration and fibrosis 11, tubular atrophy, interstitial lymphatic and mononuclear cell infiltration and fibrosis. The degree of tubulointerstitial fibrosis has been associated with the severity of renal impairment and the patient prognosis in both disease states 12-14. However, whether tubulointerstitial fibrosis is more severe in IgAN patients than in HSPN patients is unknown.

The causes of tubulointerstitial fibrosis are not entirely clear; however the association between transforming growth factor β1 (TGF-β1) and monocyte chemoattractant protein-1 (MCP-1) expression and the development of tissue fibrosis is well documented 15. TGF-β1 is a cytokine with powerful stimulatory effects on collagen production *in vivo*. Hypoxia, high serum glucose and high plasma protein levels stimulate TGF-β1 expression in human proximal tubular epithelial cells 16 and have been linked to the development of tubulointerstitial fibrosis 8,17. MCP-1 is a chemotactic factor that activates monocytes and macrophages and mediates the interstitial inflammation that precedes fibrosis. However, no reports have compared TGF-β1 or MCP-1 expression in the renal tissues from children with HSPN and IgAN.

We examined renal tissues from children with HSPN and IgAN and determined the association between TGF-β1 and MCP-1 expression and tubulointerstitial fibrosis.

## METHODS

### Study Subjects

Fifty children with HSPN and 50 children with IgAN were admitted to the Department of Nephrology, The Children's Hospital, Zhejiang University School of Medicine Hospital, between May 2012 and April 2014. The patients had similar clinical presentations with proteinuria and hematuria. The patients underwent rheumatic and anti-neutrophil cytoplasmic antibody testing to exclude vasculitis and autoimmune diseases. The HSPN diagnosis was based on the criteria recommended by the Nephrology Group of the Chinese Medical Society 18 in November 2000. A total of 32 males and 18 females with HSPN were enrolled with a mean age of 8.3±2.5 years (range: 4–13 years). The IgAN diagnosis was based on the primary glomerular nephropathy classification criteria recommended by the World Health Organization (WHO) in 1995 18. A total of 34 males and 16 females with IgAN were enrolled with a mean age of 8.8±2.1 years (range: 4–15 years). The two diagnostic groups had similar age and gender distributions. Light microscopic, immunofluorescence and electron microscopic examinations of the renal tissues were performed to confirm all diagnoses (data not shown). Patients with kidney dysfunction secondary to other diseases were excluded from this study. Informed consent was obtained from the parents of all patients. The study protocol was approved by the local institutional ethnics committee and was in accordance with the tenets of the Declaration of Helsinki. Written informed consent was obtained from all study participants after the nature of the study was explained. Clinical Trial registration number: ZJCH-2012-0105.

### Renal Tissue Collection

All patients underwent ultrasound guided renal biopsy. Two renal cores were collected from each patient. Each specimen was 1 cm to 2 cm in length. The tissues were fixed with neutral formalin solution for 24 hr and embedded in paraffin. One specimen underwent hematoxylin and eosin (H&E) and Masson’s trichrome staining for the pathological diagnosis, and the second specimen was used for TGF-β1 and MCP-1 immunohistochemical testing.

### Glomerular Pathological Classification

The HSPN glomerular findings were graded according to the International Study of Kidney Disease in Children (ISKDC) classifications as follows: grade I, minimal glomerular abnormalities; grade II, mesangial proliferation without crescents; grade III, focal segmental (IIIa) or diffuse (IIIb) mesangial proliferation with less than 50% crescents; grade IV, mesangial proliferation with 50 to 75% crescents; grade V, mesangial proliferation with greater than 75% crescents; and grade VI, membranoproliferative-like lesions.

IgAN was classified into five grades as follows: grade I, mostly normal glomeruli under the light microscope and mild mesangial proliferation without cellular proliferation; grade II, mild mesangial widening and mesangial cell proliferation with no more than three mesangial cells in each mesangial area; grade III, focal segmental glomerulonephritis with less than 50% focal or segmental glomerular, mesangial cell proliferation, and minor lesions with occasional adhesions and small crescents in the remaining glomeruli; grade IV, diffuse mesangial proliferative glomerulonephritis with significant glomerular diffuse mesangial proliferation and sclerosis and varying degrees of cell proliferation, common abandoned glomeruli, and adhesions and crescents in less than 50% of the glomeruli; and grade V, diffuse sclerosing glomerulonephritis in more than 80% of the glomeruli and crescents in more than 50% of the glomeruli 19.

### Grading of Tubulointerstitial Fibrosis

**Supplementary Figure 1:**
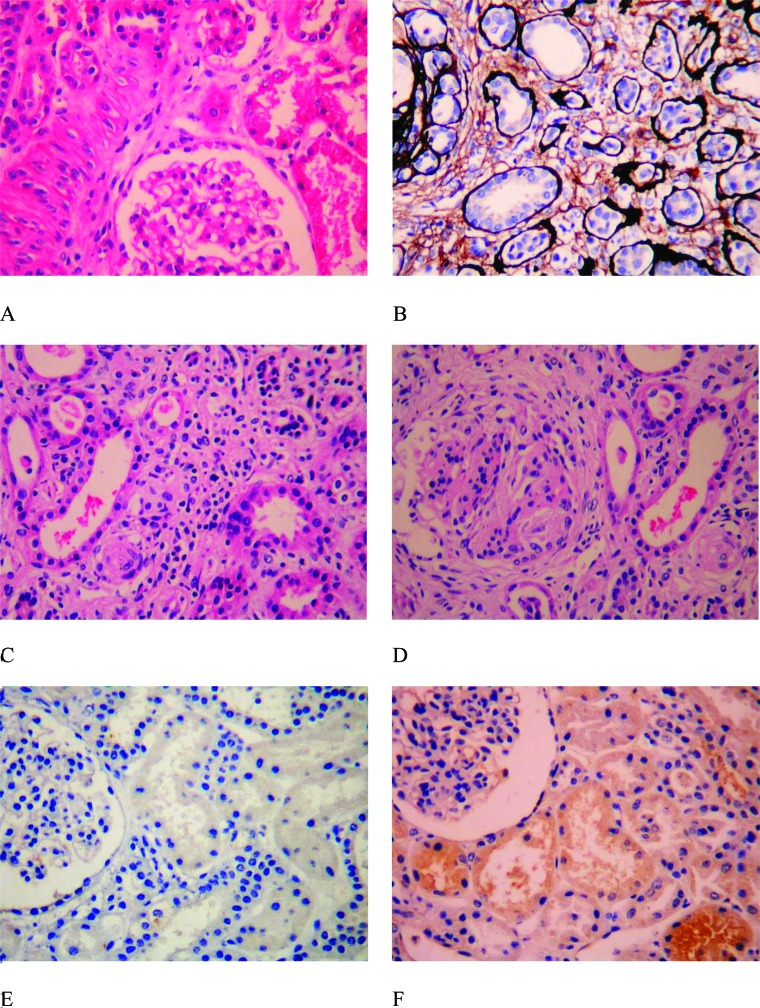

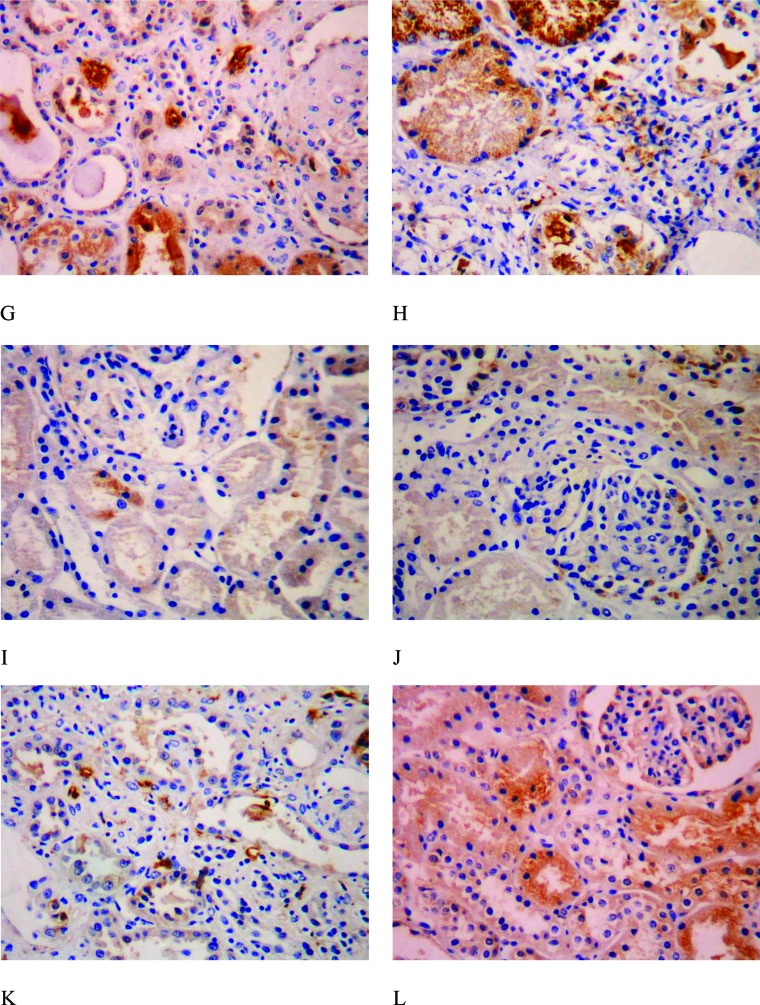
Grading of fibrosis and protein expression in renal tissues. Panels A, B, C and D show tubulointerstitial fibrosis grades +, ++, +++ and ++++, respectively. Panels A, C, and D were stained with hematoxylin and eosin, and Panel B was stained with Masson’s trichrome stain. Panels E, F, G and H show the immunohistochemical expression of TGF-β1 in the renal tubular cytoplasm of patients with IgAN. The staining intensity was -, +, ++ and +++, respectively. Panels I, J, K and L show the immunohistochemical expression of MCP-1 in the renal tubular cytoplasm of patients with HSPN. The staining intensity was -, +, ++ and +++, respectively. The magnification for all slides was 200×.

An experienced pathologist reviewed the H&E and Masson’s trichrome-stained tissues (Supplementary Figure 1A–D) to grade the level of tubulointerstitial fibrosis. Tubulointerstitial fibrosis was graded using the method described by Bohle et al. 1. Grade + was defined as normal tubulointerstitial tissue with mild tubular degeneration, grade ++ as tubulointerstitial fibrosis with less than 20% tubular atrophy and scattered inflammatory cell infiltrates, grade +++ as tubulointerstitial fibrosis with 30 to 40% tubular atrophy and scattered and/or diffuse inflammatory cell infiltrates, and grade ++++ as tubulointerstitial fibrosis with more than 50% tubular atrophy and scattered and/or diffuse inflammatory cell infiltrates 1.

### Renal TGF-β1 and MCP-1 Expression

Immunohistochemical (IHC) staining of paraffin sections was performed using the EnVision^TM^ + Kit (Dako, Denmark) according to the manufacturer’s instructions. Briefly, 1) the paraffin sections were dewaxed in xylene and then rinsed in graduated absolute ethanol solutions (95%, 80%, and 70% ethanol) and water. 2) The sections were washed with distilled water. 3) Antigen retrieval was performed using high temperature and high pressure for 100 sec in a 0.01 M (pH 6.0) citrate buffer solution. 4) The sections were washed with distilled water and then with PBS 3 times for 5 min per wash. 5) Endogenous peroxidase was blocked using 3% H_2_O_2_ for 10 min. 6) The tissue sections were washed with PBS 3 times for 5 min per wash. 7) An appropriately diluted primary antibody was added to the sections and incubated at 37°C for 1 hr (TGF-β1 (Santa cruz, California, American) at a 1:75 dilution and MCP-1 (Abcam, Cambridge, England) at a 1:100 dilution) and then at 4°C overnight. The substitution of PBS for the primary antibody was used as a negative control. 8) The slides were washed in PBS 3 times for 5 min per wash. 9) A secondary antibody (i.e., goat anti-rabbit IgG antibody-HRP multimers) was added to the sections and incubated at 37°C for 40 min. 10) The slides were washed in PBS 3 times for 5 min per wash. 11) Color development was performed using a DAB chromogenic agent for 1–3 min; the staining intensity was controlled under microscopic observation. The reaction was terminated by washing the slide with tap water. 12) The slides were stained using the Harris hematoxylin nuclear stain for 1 min and mounted with neutral resin.

TGF-β1 expression was observed in the cytoplasm of the renal tubular epithelial cells, some interstitial inflammatory cells, glomerular endothelial cells, mesangial cells and epithelial cells. MCP-1 was expressed in cytoplasm of the glomerular endothelial cells, mesangial cells, epithelial cells, tubular epithelial cells, and endothelial cells of small interstitial blood vessels. The appearance of yellow or brown particles in the cytoplasm after IHC staining was considered positive expression. The expression levels were compared to the negative controls. Expression was graded according to the scope and intensity of the staining as follows 20: negative (−), no positive staining (Supplementary Figure 1E, 1I); weak positive (+), occasional positive staining with pale yellow in less than 25% of the cells (Supplementary Figure 1F, 1J); positive (++), dark yellow staining in 26% to 50% of the cells (Supplementary Figure 1G, 1L); and strong positive (+++), brown staining in more than 50% of the cells (Supplementary Figure 1H, 1K).

### Statistical Analysis

Normally distributed data were expressed as the mean ± standard deviation. Non-normally distributed data were expressed as the median. Fisher's exact test, the Chi-square test or a T test were used for comparisons between groups. The Mann-Whitney rank sum test was used to compare the TGF-β1 and MCP-1 expression levels between the two diagnostic groups. The Spearman rank correlation analysis was used to evaluate correlations between the TGF-β and MCP-1 expression levels and the grade of tubulointerstitial fibrosis. The SPSS 11.5 software was used for the statistical analysis. A *p* value less than 0.05 was considered statistically significant.

## RESULTS

### Evaluation of Demographic Findings in Patients with HSPN and IgAN

The clinical features and intensity of proteinuria in 50 children with HSPN and 50 children with IgAN are summarized in Tables 1 and 2. No differences were observed in the clinical findings of the two groups of patients (*p*=0.18; Table 1), but a significant difference was detected in the proteinuria intensity (*p*=0.026; Table 2).

### Evaluation of Tubulointerstitial Fibrosis

The IgAN patients had significantly more severe tubulointerstitial fibrosis than the HSPN patients (*Pp*<0.001; Table 3) (Supplementary Figure 1A–D).

### Correlation between the Tubulointerstitial Fibrosis Grade and the Glomerular Findings in Children with HSPN and IgAN

The tubulointerstitial fibrosis grade was moderately correlated with the glomerular findings in both disease groups (HSPN, Spearman correlation coefficient=0.55, *p*<0.001; IgAN, Spearman correlation coefficient=0.63, *p*<0.001) (Table 4).

### TGF-β1 and MCP-1 Expression in HSPN and IgAN Patients

**Figure 1:**
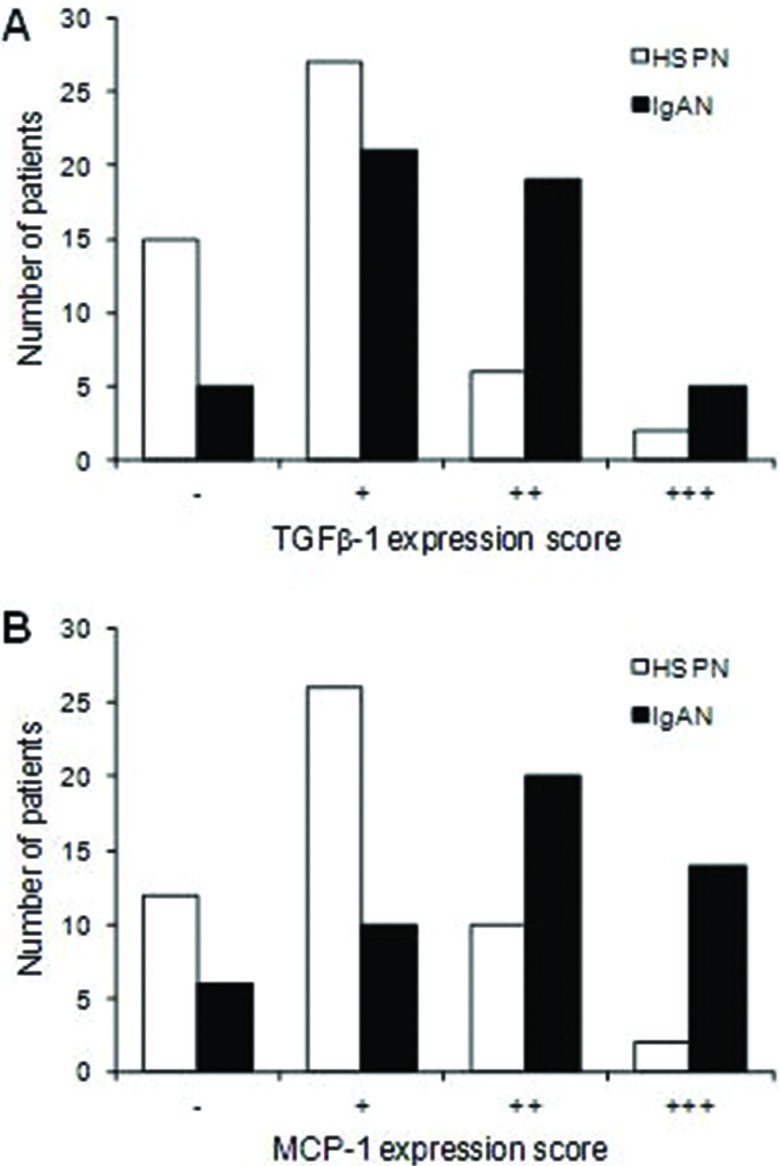
Immunohistochemical staining scores for TGF-β1 (A) and MCP-1 (B) in patients with HSPN and IgAN. The staining scores were compared between the two groups of patients with the Mann–Whitney–Wilcoxon test. Z=3.6013, *p*=0.0003 for TGF-β1 and Z=3.94, *p*<0.001 for MCP-1.

TGF-β1 and MCP-1 expression was significantly greater in the IgAN patients than in the HSPN patients (*p*<0.001 for both) (Figure 1).

### Correlation between the Tubulointerstitial Fibrosis Grade and TGF-β1 and MCP-1 Expression

The tubulointerstitial fibrosis grade was moderately correlated with TGF-β1 and MCP-1 expression in both disease groups (HSPN: Spearman correlation coefficient for TGF-β1=0.56, *p*<0.001; Spearman correlation coefficient for MCP-1=0.63, *p*<0.001; IgAN: Spearman correlation coefficient for TGF-β1=0.72, *p*<0.001; Spearman correlation coefficient for MCP-1=0.62, *p*<0.001) (Tables 5–8).

## DISCUSSION

Increased TGF-β1 and MCP-1 expression has been reported in many fibrotic diseases 21. TGF-β1 is a cytokine that is expressed in the kidney in association with the development of tubulointerstitial fibrosis 22. MCP-1 plays a role in the chemotaxis and activation of monocytes and macrophages. Tubulointerstitial injury is related to monocyte and macrophage infiltration, which stimulates the ingrowth of activated neutrophils, mononuclear cells, and subsequent T cell infiltration and in turn stimulates the growth of fibroblasts and keratinocytes 23. MCP-1 also stimulates the expression of IL-6, cell adhesion molecules, and other inflammatory factors that contribute to renal tubular fibrosis 24.

TGF-β1 has been hypothesized to participate in the initiation and progression of early immune renal tubular injury and the formation of tubulointerstitial fibrosis 25. Yamumoto et al. 17. examined renal tissues from patients with different types of renal disease. Patients with thin basement membrane nephropathy or minimal change nephropathy had TGF-β1 mRNA expression levels that were similar to the normal control patients. Conversely, patients with IgAN, HSPN, lupus nephritis or crescent nephritis and glomerular or tubulointerstitial disease had significantly greater renal TGF-β1 mRNA expression levels than the normal control patients, which was consistent with our findings. We found that the IHC expression of TGF-β1 in the renal tubular epithelial cells was positively correlated with the tubulointerstitial fibrosis grade in children with IgAN. Together, these findings suggest that renal TGF-β1 expression may be related to tubulointerstitial disease and impaired renal tubular functions in IgAN patients.

A study of 25 adult IgAN cases by Celie et al. 26 demonstrated an association between MCP-1 expression and the degree of glomerular and tubulointerstitial injury. MCP-1 expression in the tubulointerstitial tissues was positively correlated with the amount of proteinuria. MCP-1 expression in the glomerular and tubulointerstitial tissues was suggested to be a possible indicator of renal disease severity in IgAN patients. We found a positive correlation between MCP-1 expression and the grade of tubulointerstitial fibrosis. Together, these findings suggest that MCP-1 expression is related to the tubulointerstitial fibrosis and impaired tubular function found in IgAN patients.

Patients with HSPN typically present with acute episodes of glomerular inflammation consisting of mesangial proliferation, fibrin deposits and epithelial crescents that heal spontaneously or lead to chronic lesions. Sick children with HSPN suffer from humoral immune abnormalities, T cell subset dysfunction, and abnormal cytokine production. These findings may lead to TGF-β1 expression, which can mediate fibrosis formation. In contrast, patients with IgAN typically have an IgA-based granular sediment deposited in the glomerulus mesangial region. These patients develop slowly progressive mesangial lesions from the continuous low-grade deposition of macromolecular IgA1. Abnormal IgA1 glycosylation in these patients is associated with renal deposition and complement system activation. The children with HSPN examined in this study had low renal TGF-β1 and MCP-1 expression, whereas the IgAN patients commonly had high expression levels of these cytokines. The mechanism of inflammation initiation appears to be more active in patients with IgAN, which may explain why these patients have more fibrosis and higher TGF-β1 and MCP-1 expression levels than the HSPN patients.

Increased renal TGF-β1 and MCP-1 expression was associated with increased tubulointerstitial fibrosis in both disease states. Tubulointerstitial fibrosis was observed in both diagnostic groups but was more common in the IgAN group. We found a similar positive moderate correlation between the tubulointerstitial fibrosis grade and the intensity of proteinuria and TGF-β1 and MCP-1 expression in both diseases. The relative frequency of cytokine expression suggests that TGF-β1 and MCP-1 are likely linked to tubulointerstitial fibrosis formation in both disease groups. Several studies have shown that higher proteinuria levels are related to the progression of renal diseases in patients with different types of glomerulopathy 27, which is consistent with the results of our study (Table 2). One possible mechanism is that the increase in protein reabsorption by the tubule cells leads to changes in these cells, which start to produce cytokines and chemoattractant factors for lymphocytes and macrophages; then, these cells migrate to the interstitial area to induce inflammation and fibrosis 28-30. Further work is needed to better understand the development of inflammation and TGF-β1 and MCP-1 expression in these patients and the progression to tubulointerstitial fibrosis.

In summary, we observed tubulointerstitial fibrosis in the renal tissues of children with HSPN and IgAN. Greater tubulointerstitial fibrosis was observed in the IgAN patients compared to the HSPN patients. Increased amounts of tubulointerstitial fibrosis were associated with increased TGF-β1 and MCP-1 expression. These findings suggest that TGF-β1 and MCP-1 may be involved in the development of tubulointerstitial fibrosis in both these disease states, possibly through different inflammatory mechanisms.

## AUTHOR CONTRIBUTIONS

All authors were involved in drafting the manuscript or critically revising it for important intellectual content, and all authors approved the final version for publication. Jianhua M has full access to all data in the study and takes responsibility for the integrity of the data and the accuracy of the data analysis. Shuiai Z and Jianhua M conceived and designed the study and were responsible for the data analysis and interpretation. Shuiai Z, Huijun S and Weizhong G were responsible for the data acquisition. Aimin L and Jianhua M were responsible for the clinical data collection and patients diagnosis.

## Figures and Tables

**Table 1:** Clinical findings in patients with HSPN and IgAN.

Clinical finding	HSPN		IgAN		χ^2^	*p*
	n	%	n	%		
Proteinuria	46	92%	48	96%	4.89	0.18
Hematuria and proteinuria	25	50%	20	40%		
Acute nephritis	5	10%	10	20%		
Nephrotic syndrome	10	20%	5	10%		
Rapidly progressive glomerulonephritis	0		0			

**Table 2:** Intensity of proteinuria in patients with HSPN and IgAN.

24 h proteinuria	HSPN		IgAN		Z	*p*
	n	%	n	%		
<25 mg/kg	19	41.3%	8	16.7%	2.23	0.026
25-50 mg/kg	17	36.9%	25	52.1%		
50 mg/kg	10	21.7%	15	31.2%		

**Table 3:** Grading of tubulointerstitial fibrosis in patients with HSPN and IgAN.

Fibrosis grade	HSPN	IgAN
I	38	5
II	9	17
III	2	20
IV	1	8
Total	50	50

Z=6.74, *p*<0.001, by Mann–Whitney–Wilcoxon test.

**Table 4:** Correlation between the tubulointerstitial fibrosis grade and the glomerular pathological findings in patients with HSPN and IgAN.

Disease grade	Grade of fibrosis in HSPN^a^			Grade of fibrosis in IgAN^b^		
	I–II	III	IV–V	I–II	III	IV–V
I	26	12	0	4	1	0
II	2	6	1	13	3	1
III	0	0	2	3	13	4
IV	0	0	1	1	8	5
V	0	0	0	0	0	0

a χ^2^=21.44, *Pp*<0.001 for children with HSPN, and ^b^χ^2^=17.83, *p*<0.001 for children with IgAN.

**Table 5:** Correlation between the tubulointerstitial fibrosis grade and MCP-1 expression in children with HSPN.

Fibrosis grade	n	MCP-1 expression			
		-	+	++	+++
I	38	12	23	3	0
II	9	0	3	6	0
III	2	0	0	1	1
IV	1	0	0	0	1
Total	50	12	26	10	2

Spearman correlation coefficient for MCP-1=0.63, *p*<0.001.

**Table 6:** Correlation between the tubulointerstitial fibrosis grade and TGF-β1 expression in HSPN.

Fibrosis grade	n	TGF-β1 expression			
		-	+	++	+++
I	38	15	21	2	0
II	9	0	6	3	0
III	2	0	0	1	1
IV	1	0	0	0	1
Total	50	15	27	6	2

Spearman correlation coefficient for TGF-β1=0.56, *p*<0.001.

**Table 7:** Correlation between the tubulointerstitial fibrosis grade and MCP-1 expression in IgAN.

Fibrosis grade	n	MCP-1 expression			
		-	+	++	+++
I	5	4	1	0	0
II	17	2	7	4	4
III	20	0	2	14	4
IV	8	0	0	2	6
Total	50	6	10	20	14

Spearman correlation coefficient for MCP-1=0.62, *p*<0.001.

**Table 8:** Correlation between the tubulointerstitial fibrosis grade and TGF-β1 expression in IgAN.

Fibrosis grade	n	TGF-β1 expression			
		-	+	++	+++
I	5	3	2	0	0
II	17	2	11	4	0
III	20	0	8	12	0
IV	8	0	0	3	5
Total	50	5	21	19	5

Spearman correlation coefficient for TGF-β1=0.72, *p*<0.001.
